# Environmental, financial, and macroeconomic determinants of life expectancy in E7 economies

**DOI:** 10.3389/fpubh.2026.1850171

**Published:** 2026-07-01

**Authors:** Jianhui Guo, Yingqi Su, Xiaofen Li, Peiyan Wu

**Affiliations:** 1 School of Economics, Guangxi University, Guangxi, Nanning, China; 2 College of Business, Nanning University, Guangxi, Nanning, China; 3 School of Business, Guangxi University, Guangxi, Nanning, China

**Keywords:** E7 economies, financial development, healthcare infrastructure, macroeconomic stability, PM2.5, public health outcomes

## Abstract

This paper analyzes the long-run associations between environmental, financial, macroeconomic, and healthcare factors on life expectancy in E7 countries over the period 1990–2023. Special emphasis is placed on PM2.5 air pollution exposure, which has become one of the key environmental factors influencing population health and longevity. The analysis examines the effects of domestic credit to the private sector (DCPS), foreign direct investment (FDI), inflation (INF), exposure to PM2.5 air pollution (PM2.5), immunization coverage (IMM), and hospital beds per 1,000 people (HBEDS) using life expectancy (LE) as the dependent variable. Second-generation panel unit root and cointegration tests are used in the empirical framework. FMOLS and DOLS estimators are then used. The CCEMG estimator and FE-DKSE are used to evaluate robustness. The findings show a stable long-run connection between the variables. Financial development and FDI are positively associated with LE, but inflation and PM2.5 exposure are negatively associated with LE. Immunization coverage is generally positively associated with life expectancy, while hospital beds are negatively associated with LE. The robustness estimations generally support the baseline findings. This study adds to existing research by examining how finances, economy, environment, and health care impact LE in the E7 countries. It takes into account the connections and differences between these nations. The findings highlight that financial growth, economic stability, clean environments, and preventive healthcare play crucial roles in long-term health outcomes for large emerging economies.

## 1 Introduction

Health outcomes and economic development go hand in hand in sustainable progress nowadays. When people are healthier, they are more productive and easier to integrate into the workforce, which in turn boosts the economy (1). Plus, an improved economy helps pay for better healthcare and living standards. However, for developing countries, rapidly growing economically can complicate things. They must handle financial shifts, deal with environmental issues, and support growing healthcare needs (2). This all makes figuring out what affects public health super important to both researchers and policymakers.

In the global economy, the E7 countries, India, China, Brazil, Mexico, Russia, Turkey, and Indonesia, are super important in emerging markets. They have a big chunk of the world’s population and economy, along with lots of industrial action and energy use. So, they play a massive role globally (3). Plus, they are predicted to become more influential in global growth over the next few decades (4). However, these nations also grapple with major public health issues due to fast-paced urbanization, a worsened environment, healthcare disparities, and shifts in the economy. Because of this, studying the E7 provides a unique way to look at how economics, the environment, and healthcare all affect the health of their populations (5).

Health outcomes are impacted by economic factors in a number of ways. Financial growth, for example, facilitates loan applications, increases investment, and makes it possible for consumers and governments to more efficiently fund healthcare. Therefore, improved health care is typically associated with economic prosperity (6). Moreover, foreign direct investment (FDI) brings new tech, creates jobs, builds up infrastructure, and boosts living standards (7). However, inflation hits hard too – it wrecks purchasing power, drives up health care costs, and weakens both public and private health spending (8). The quality of the environment has become one of the key factors affecting population health, especially when referring to countries experiencing rapid industrialization (9, 10). In terms of environmental health hazards, fine particulate matter (PM2.5) exposure has been established as one of the primary contributors to premature death in today’s world. Due to its minute size, PM2.5 exposure poses several serious health concerns due to the ability of particles to deeply affect the respiratory system and penetrate blood circulation, thereby leading to heart disease, stroke, lung disease, and premature death (11, 12). There is sufficient evidence showing that prolonged exposure to PM2.5 is associated with severe effects on longevity and considerable health-related costs. Taking into account rapid industrialization, increasing urbanization, and growing energy use in the context of the E7 countries, it becomes important to analyze the importance of PM2.5 exposure as the key determinant of population health rather than just a control variable. Consequently, environmental quality constitutes a fundamental component of long-term health and sustainable development in emerging economies (13).

The factors influencing health outcomes in the E7 economies are still not well understood, even though these economies keep growing in importance. Most past research focuses on developed nations, looking mainly at single factors like health spending and income. Yet, existing studies rarely examine how financial growth, foreign investment, macroeconomic balance, and environmental quality interact over time to impact LE. Additionally, a lot of these studies look at each factor independently and often ignore complexities such as cross-sectional dependence, country-specific features, and long-term equilibrium relationships. Therefore, we still have big questions about how crucial financial, macroeconomic, environmental, and healthcare factors are in shaping health results across major emerging economies.

In light of this, the current study looks at the long-term relationships between financial development, foreign direct investment, inflation, exposure to PM2.5 air pollution, vaccination rates, healthcare infrastructure, and LE in the E7 economies between 1990 and 2023. Because life expectancy reflects the combined effects of social development, healthcare access, environmental quality, and economic conditions, it is used as the main indicator of population health. The study offers a more thorough evaluation of the variables linked to health outcomes in significant emerging economies by combining financial, macroeconomic, environmental, and health-system determinants into a single empirical framework.

This study makes several significant contributions to the literature. By studying financial development, foreign direct investment, inflation, environmental quality, preventative healthcare, and healthcare infrastructure all at once within a single framework, it first expands on previous research. Second, it provides fresh data on the economies of the E7, a group of nations that is becoming more and more important on a global scale but is still mostly unexplored. Third, the study uses a thorough econometric approach that uses numerous cointegration tests, different long-run estimators, and second-generation panel approaches to account for cross-sectional dependency, slope heterogeneity, non-stationarity, and endogeneity. As a result, the study offers stronger proof of the long-term factors influencing LE than a large portion of the current body of research.

This study looks into some research questions, too. First, do domestic credit to the private sector, foreign direct investment, inflation, PM2.5 air pollution, immunization coverage, and hospital beds affect LE in the E7 economies from 1990 to 2023? Also, how much do financial development, foreign investment, environmental quality, preventive health care, and health care infrastructure boost LE in those countries? Lastly, do the findings still hold up when considering cross-sectional dependence, country differences, and common shocks in emerging economies?

## 2 Literature review

### 2.1 Financial development and health outcomes

Grossman’s (14) Health Capital Theory, which sees health as a type of capital that people invest in to improve productivity, well-being, and longevity, provides the foundation for the link between financial development and health outcomes. By increasing credit availability, encouraging savings, lowering liquidity constraints, and enhancing resource allocation within the economy, financial development can support these initiatives (15). Households are better equipped to pay for preventative services, healthcare costs, consuming a nutritious diet, and other activities that improve health, and recognition of these mechanisms.

On a bigger scale, financial development aids economic growth, creates jobs, and increases incomes, making people healthier. When finance runs smoothly, there’s more money for important things like healthcare. Studies show that solid finances lead to healthier people and improved overall well-being. Mbodj and Laye (16) discovered that financial development improves social welfare and human development, which includes population health. Miar (17) also points out that economic stability enhances general well-being and development, including health. Research by Jalili et al. (18) shows that with better financial resources, families can manage health crises better and access necessary care more easily, helping them live longer. Both theory and real-world data suggest that financial growth should enhance health outcomes in developing countries.

### 2.2 Foreign direct investment and health outcomes

Modernization Theory says that external capital spurs tech advances, boosts productivity, builds up institutions, and reshapes the economy (19). This theory also shows how foreign direct investment is linked to better health. FDI improves the social and economic environments that promote health, raising living standards in the process.

Foreign investment affects health in various ways, too. It spurs economic growth and creates jobs, which boosts people’s incomes and access to healthcare. Plus, multinational firms bring in new tech and management methods. This not only makes industries more efficient but also pushes forward development goals like health projects. So, it’s clear that foreign investment matters for improving health (20). Lastly, when FDI drives up economic activity, governments collect more taxes and can invest more in healthcare and social programs.

Empirical studies mostly back up FDI’s positive effects on welfare and human development. Zeeshan and Singh (21) point out that foreign investment aids economic modernization and social progress in developing countries. Similarly, Nguyen et al. (22) found that FDI boosts development via better economic performance and increased investment capacity. Yet, the benefit size relies on things like institutional quality, how well a country is run, and its absorptive ability (23). So overall, research suggests FDI is probably linked to longer life expectancies.

### 2.3 Inflation, macroeconomic stability, and health outcomes

Long-term gains in population health need macroeconomic stability. Welfare economics shows that steady prices shield household purchasing power, making healthcare more accessible, which helps societies get the medical care they need. On the other hand, prolonged inflation can negatively impact health outcomes in a number of ways (24). Prices going up drive costs for medical care, drugs, and healthy food way higher, making these things much harder to afford – especially for those on lower incomes (25). Inflation drives up healthcare costs for the government, leaving less money for other health programs (26). It also throws job markets and income predictability out of whack, messing with household stability (27).

Plus, Pappas and Boukas (28) show that macroeconomic volatility hurts social welfare and development. So, inflation seriously impacts health outcomes. In a similar vein, Manamperi et al. (29) note that inflationary pressures impair gains in population health and decrease access to healthcare. According to other research, persistent inflation can lower living standards and impede the advancement of health indicators, especially in developing and rising nations with potentially underdeveloped social protection systems (30). As a result, it is anticipated that inflation will reduce LE.

### 2.4 Environmental quality and health outcomes

Particularly in nations that are industrializing quickly, environmental quality has become a crucial factor in determining population health (31). The Environmental Health Risk Framework, which highlights how environmental exposures directly impact morbidity and mortality through biological and physiological processes, is frequently used to describe the relationship between environmental conditions and health (32).

It’s widely known that one of the greatest threats to public health from environmental hazards is exposure to PM2.5. These particles are super small, so they can go deep into the lungs and even enter the bloodstream. This raises the risk of heart issues, strokes, and other serious respiratory problems – even death (33). On a global scale, constant exposure to high PM2.5 levels is tied to big drops in life expectancy and hefty public health expenses (34).

Empirical evidence clearly shows how harmful air pollution is to health. Henning (35) demonstrated that worse air quality leads to greater health risks, especially in developing and emerging economies. Many other studies show that decreasing exposure to PM2.5 reduces mortality rates and boosts life expectancy (36).

Rapid industrialization, urban growth, and increased energy use in the E7 countries make preserving a healthy environment really important for people’s long-term well-being (37). Higher PM2.5 levels likely mean shorter lifespans and lower life expectancy, too.

### 2.5 Health-system capacity and population health

Health outcomes rely on economic and environmental factors, as well as how well our healthcare systems can stop sickness and offer good care (38). This is backed by Health Production Theory, showing that health is created through important parts of the healthcare system (39).

Preventive measures really matter because they lower illness and death rates. Immunization programs stand out as a huge win for public health; they have stopped infections and reduced deaths among kids (40). Not only do high vaccination rates show effective health service delivery, but they also highlight strong public health initiatives that protect everyone’s wellness (41).

Healthcare infrastructure is another key factor for health outcomes. Hospital beds reflect a health system’s ability to handle patient needs (42). Enough healthcare facilities make the system more ready when lots of people need care, making medical services easier to access (43).

Empirical studies highlight the crucial role of preventive healthcare and medical systems in boosting public health. Huang et al. (44) demonstrate that successful immunization programs lead to major health improvements and help stop diseases. Likewise, Vărzaru (45) and Sanchez Leitner et al. (46) found that solid healthcare infrastructure means better health results and higher life expectancy. Accordingly, immunization coverage and healthcare infrastructure are expected to contribute positively to life expectancy.

The following hypotheses are put forth in light of the theoretical underpinnings and empirical data previously discussed:

*H1*: In the selected economies, life expectancy is positively correlated with domestic lending to the private sector.

*H2*: In the E7 economies, life expectancy is positively correlated with foreign direct investment.

*H3*: In the selected nations, life expectancy is inversely correlated with inflation.

*H4*: In the E7 economies, exposure to PM2.5 air pollution has a negative correlation with life expectancy.

*H5*: In the study region, life expectancy is favorably correlated with immunization coverage.

*H6*: In the E7 economies, life expectancy is positively correlated with healthcare infrastructure as assessed by hospital beds per 1,000 people.

### 2.6 Research gap

Despite making great strides in understanding what affects health outcomes, there are still some big gaps in the research. First off, studies tend to focus on financial growth, foreign investment, economic stability, environmental quality, and health care capacities separately, not how they mesh together to affect overall health. Another key problem is the lack of specific research on the E7 economies. These nations have an enormous portion of the global population, economic output, energy consumption, and environmental effects. With their rapid development and growing economic clout, understanding what drives their health results could really benefit both academics and policymakers.

Thirdly, while a considerable number of studies have shown that PM2.5 exposure is one of the important determinants for increased mortality and decreased life expectancy, fewer studies have considered its relationship with health over a long period of time and the interaction between financial development, macroeconomic variables, and health care characteristics in major emerging economies. Hence, it seems that the environmental and health consequences of fast economic growth in the group of E7 economies have been understudied. Moreover, most of the studies focus on individual analysis of the factors in question instead of a holistic approach and seldom undertake a long-term panel data analysis. Previous research tends to use standard panel data approaches without taking into account cross-sectional dependence associated with globalization, financial integration, and common shocks affecting multiple countries simultaneously. Furthermore, despite significant variations in institutions, healthcare systems, and development paths among rising economies, country-specific reactions to comparable economic, environmental, and healthcare factors are frequently disregarded.

To address these issues, our study explores the long-term links between domestic credit to the private sector, foreign direct investment, inflation, PM2.5 air pollution, immunization rates, healthcare infrastructure, and life expectancy in the E7 economies from 1990 to 2023. We look at financial, macroeconomic, environmental, and health factors all in one comprehensive model. Plus, we use an empirical approach that tackles cross-sectional dependence and slope heterogeneity, making our analysis stronger.

Our analysis relies on second-generation panel unit root tests like CIPS and CADF, several panel cointegration tests including Pedroni, Kao, and Westerlund, and long-run estimators such as FMOLS and DOLS. For extra assurance, we double-check results with fixed effects and Driscoll-Kraay standard errors, and the Common Correlated Effects Mean Group (CCEMG) estimator. Altogether, this lets us reliably assess long-term relationships while handling non-stationarity, endogeneity, common shocks, and varying country responses.

As a result, this work makes two primary contributions. First, it offers a comprehensive evaluation of the macroeconomic, financial, healthcare, and environmental factors that affect life expectancy in the E7 economies, a topic that has not received much attention in the literature so far. Second, it makes a methodological contribution by utilizing a thorough econometric framework that ensures robust and dependable inference by combining numerous cointegration approaches, second-generation panel methodologies, and alternative long-run estimators. By doing this, the study strengthens the empirical basis for the development of policies targeted at enhancing long-term health outcomes and presents fresh information on the variables linked to population health in significant rising economies.

## 3 Data and methodology

### 3.1 Data

The current study analyzes the factors affecting life expectancy in the group of the E7 countries that consist of China, India, Brazil, Mexico, Russia, Indonesia, and Turkey between 1990 and 2023. The sample period was selected based on the availability of consistent and comparable annual data for all countries and variables included in the analysis. The selected group of countries is considered one of the biggest and fastest-growing emerging markets, and they cover a large share of both the world population and economy. Therefore, it makes perfect sense to consider them in terms of finding the link between the level of economic development, environment, and healthcare system, and life expectancy in the long run. For all variables mentioned above, the data were taken from the World Development Indicators (WDI), which is a part of the World Bank. Linear interpolation was used for filling in the missing observations for constructing the full panel without losing time-series features of each variable. This approach allowed the construction of a complete dataset while preserving the underlying trends and characteristics of the original series. Details on the interpolation process, involving the variables, countries, and years involved in the interpolation, are presented in Appendix Table A1.

Table 1 illustrates the variables that were utilized in this research. Life expectancy at birth (LE) serves as the dependent variable in this study due to its comprehensiveness as well as popularity as an indicator of population health status. Life expectancy at birth represents the total of all health-related influences, ranging from healthcare accessibility, disease prevention, environment, living standards, nutrition, and general socioeconomic progress. Unlike disease-specific indicators, it captures the overall health status of a population and therefore provides a suitable measure for evaluating the long-run determinants of health across countries.

**Table 1:** Variable description.

Variable name	Abbreviation	Description	Source
Life expectancy at birth, total (years)	LE	Average number of years a newborn is expected to live, assuming mortality patterns at the time of birth remain constant.	WDI (57)
Foreign direct investment, net inflows (% of GDP)	FDI	Net inflows of investment to acquire a lasting management interest in an enterprise operating in the economy as a share of GDP.	WDI (57)
Domestic credit to private sector (% of GDP)	DCPS	Financial resources provided to the private sector by banks and other financial institutions as a share of GDP.	WDI (57)
Immunization, measles (% of children ages 12–23 months)	IMM	Percentage of children ages 12–23 months who received measles immunization.	WDI (57)
PM2.5 air pollution, mean annual exposure (μg/m^3^)	PM2.5	Population-weighted exposure to fine particulate matter (PM2.5), measured in micrograms per cubic meter.	WDI (57)
Inflation, consumer prices (annual %)	INF	Annual percentage change in the cost to the average consumer of acquiring a basket of goods and services.	WDI (57)
Hospital beds (per 1,000 people)	HBEDS	Number of hospital beds available per 1,000 people, indicating healthcare capacity.	WDI (57)

The main explanatory variables in this study include domestic credit to the private sector (DCPS), inflation (INF), and PM2.5 air pollution exposure (PM2.5). DCPS represents the degree of financial development within a nation since DCPS is used as a measure of how well financial systems provide funding for activities that boost productivity, investments, and living standards. Financially developed nations will have better access to health services, better infrastructural development in the health sector, and an improved general standard of living. Inflation is used as a measure of macroeconomic stability since continuous price increases lead to increased healthcare expenditure, thereby limiting the benefits of both public and private health expenditure. Environmental quality is captured by incorporating exposure to PM2.5 air pollution. Because continuous exposure to fine particulate matter can raise the incidence of respiratory and cardiovascular disorders, contribute to early mortality, and shorten life expectancy, it is widely acknowledged as a major environmental health issue.

Moreover, the inclusion of foreign direct investment (FDI) as a control variable in the model allows for consideration of how external investment affects the economic development and well-being of a country’s population. Foreign investments can drive economic growth and contribute to technology transfers, job creation, and indirectly contribute to improved healthcare services. The model will also include immunizations against measles (IMM) and hospital beds per 1,000 people (HBEDS) as indicators of the health system’s capacity. The measure of immunizations against measles can reveal the efficiency of preventive healthcare practices and the success of efforts made to reduce mortality from preventable diseases. Finally, the measure of hospital beds per 1,000 people reflects the availability of healthcare services and facilities within healthcare systems in E7 countries. This way, the model considers multiple aspects, including finance, macroeconomic stability, environment, investment, preventive healthcare, and healthcare capacity that impact life expectancy in E7 countries.

### 3.2 Methodology

The following empirical model is described to investigate the connections among financial development, macroeconomic conditions, environmental quality, and life expectancy in the E7 economies:


$$\begin{array}{l} LE_{\mathrm{it}}=\alpha_{i}+\beta_{1}\mathrm{DCP}S_{\mathrm{it}}+\beta_{2}\mathrm{FD}I_{\mathrm{it}}+\beta_{3}\text{IN}F_{\mathrm{it}}+\\ \beta_{4}\mathrm{PM}2.5_{\mathrm{it}}+\beta_{5}\mathrm{IM}M_{\mathrm{it}}+\beta_{6}\text{HBED}S_{\mathrm{it}}+\varepsilon_{\mathrm{it}} \end{array}$$

where LE_it_ represents life expectancy, DCPS_it_ captures domestic financial development, FDI_it_ measures foreign investment inflows, and PM2.5_it_ captures environmental quality. INF_it_ reflects macroeconomic instability, IMM_it_ indicates immunization coverage, and HBEDS_it_ captures healthcare infrastructure. α_i_ represents the country effect, and β_1_−β_6_ are the coefficients to be estimated, while εit is the error term.

All variables were converted to standardized z-scores before estimation. To put the variables on a uniform scale, lessen the impact of variations in measurement units, and enhance the comparability of coefficient estimates across variables, standardization was carried out. The transformation makes it easier to understand the relative strength and direction of the factors’ correlations with life expectancy while maintaining the underlying linkages between the variables.

In order to analyze the factors determining the long-run levels of life expectancy in E7 countries, an empirical methodology is employed to undertake descriptive, diagnostic, and econometric tests in an appropriate manner. This multi-stage approach ensures the reliability of the results while accounting for the time-series and cross-sectional characteristics of the panel data.

Firstly, the descriptive statistics and trend analysis are conducted. The former provides details about the central tendency and dispersion of the variables along with the distribution of the variables (47). On the other hand, graphical techniques reveal the country-specific trends and long-term trends over time. Moreover, a correlation matrix is computed to understand the nature of the relationship between the variables in terms of theoretical expectations regarding financial development, macroeconomic performance, environmental quality, health infrastructure, and life expectancy moving together in theoretically expected ways (48).

Examining whether cross-sectional reliance exists within the panel is crucial given the E7 economies’ increasing economic interconnectedness and their vulnerability to shared global shocks. The Pesaran (49) Cross-Sectional Dependence (CD) test is therefore used. Cross-sectional reliance suggests that shocks to one nation may have an impact on other nations through international economic events, commerce, investment, or financial markets. Ignoring this relationship could result in incorrect data and biased statistical inference. Additionally, the Pesaran and Yamagata (50) slope homogeneity test is used to ascertain whether there is country-specific heterogeneity or whether the calculated connections are homogeneous across nations. Testing for slope heterogeneity is crucial before choosing suitable panel estimators because the E7 economies differ significantly in terms of institutional development, economic structure, healthcare systems, and environmental circumstances.

Once the above diagnostics are performed, the stationarity characteristics of the variables are analyzed through the use of second-generation panel unit root tests. In particular, the Cross-Sectionally Augmented Dickey-Fuller (CADF) and Cross-Sectionally Augmented IPS (CIPS) tests developed by Pesaran (51) are utilized. Such tests are used since they take into account the issue of cross-sectional dependence among panel observations and are hence better suited than first-generation tests. Determining the order of integration is extremely important as regressions with non-stationary variables have a tendency to produce spurious results if long-run equilibrium properties are not properly considered.

After establishing that the variables are of the same order of integration, the next step is to establish whether there exists a long-run equilibrium relationship between them using panel cointegration techniques. In order to provide robust evidence on the cointegration results, the following three cointegration tests will be conducted: the Pedroni cointegration test, the Kao cointegration test, and the Westerlund cointegration test. The Pedroni (52) test allows for heterogeneity in the panel and includes several within and between dimension tests. The Kao cointegration test acts as another residual-based cointegration test under the homogeneous cointegration framework. The Westerlund (53) cointegration test is especially important since it provides robust results for evaluating the existence of an error correction mechanism in the panel in the presence of cross-section dependence. The use of multiple cointegration procedures provides stronger evidence regarding the existence of a stable long-run relationship among the variables.

In the next step, after proving the existence of co-integration, the estimation of long-run coefficients will be made based on the Fully Modified Ordinary Least Squares (FMOLS) model. The reason why the FMOLS approach is popular in the literature for panel cointegration is due to its ability to control for the effect of serial correlation and endogeneity due to the presence of the co-integration relation. By making a nonparametric correction, FMOLS is able to consistently estimate long-run coefficients in the presence of endogenous regressors. Consequently, it serves as the primary estimator for evaluating the long-run association between financial development, macroeconomic conditions, environmental quality, healthcare factors, and life expectancy.

In order to test the consistency of the FMOLS estimates, Dynamic Ordinary Least Squares (DOLS) are also used. DOLS improve upon the concept of cointegration through the use of lead and lag terms of first differences of regressors to better deal with the issues of endogeneity and small sample bias. Because DOLS and FMOLS rely on different correction mechanisms, consistency between their results increases confidence in the stability and reliability of the estimated long-run relationships (54).

In addition, a panel data fixed-effects regression using Driscoll-Kraay standard errors is estimated after performing the Hausman specification test. The Hausman specification test is conducted in order to check the most appropriate panel data specification based on the correlation between the individual effects and explanatory variables. In case the fixed-effects specification is chosen, Driscoll-Kraay standard errors are considered more appropriate due to their robustness to heteroskedasticity, serial correlation, and cross-sectional dependence (55). Moreover, the application of the Driscoll-Kraay standard error method improves the accuracy of statistical inference when there are common shocks and correlations between the countries being studied, especially in the context of the highly integrated E7 countries.

In the last section, the CCEMG (Common Correlated Effects Mean Group) estimator, introduced by Pesaran (56), is applied as an additional robustness test. The CCEMG estimator takes into consideration the effects of unobservable common factors, cross-sectional dependence, and slope heterogeneity by including cross-sectional averages of the regressand and the regressors in the regression model. Since the economies of E7 are interlinked, and there could be varying country-specific responses to financial, environmental, and macroeconomic circumstances, CCEMG provides a particularly suitable framework for assessing the robustness of the estimated long-run relationships.

Indeed, the use of tests for diagnostic purposes, unit roots and cointegration techniques using generation two, FMOLS and DOLS estimation methods, fixed effects estimates with Driscoll-Kraay SEs, and the CCEMG approach gives a robust methodology for analyzing the factors influencing life expectancy in the E7 countries. This approach enhances the robustness of the analysis by addressing issues of non-stationarity, endogeneity, cross-sectional dependence, heterogeneity, and common shocks within a unified empirical framework.

## 4 Results and discussion

The descriptive data for the variables utilized in the analysis across the E7 economies from 1990 to 2023 are shown in Table 2. The findings show significant diversity in financial, macroeconomic, environmental, and healthcare-related metrics, which represent the sample countries’ varied developmental experiences. Overall, the descriptive statistics reveal a number of shared long-term trends in health outcomes, financial development, environmental quality, and healthcare infrastructure, while also offering preliminary evidence of significant variation across the E7 economies.

**Table 2:** Descriptive statistics for E7 economies.

Variable	Mean	Median	Std. Dev.	Min	Max	Skewness	Kurtosis	Jarque–Bera
LE	70.461	70.432	4.283	58.618	78.202	−0.354	2.652	6.164**
DCPS	49.529	36.308	35.669	12.241	189.607	1.843	6.114	230.8***
FDI	1.917	1.820	1.362	−2.757	6.162	0.150	3.467	3.063
INF	61.336	6.799	284.978	−1.401	2947.733	7.430	63.929	3.9E+04***
PM2.5	30.109	22.922	17.035	11.273	79.037	1.006	2.787	40.58***
IMM	86.915	90.000	12.201	43.000	99.000	−1.078	3.573	49.35***
HBEDS	3.075	2.400	2.880	0.560	13.055	1.902	5.717	216.7***

p*** < 1% and p** < 5%. Raw data were used for descriptive analysis. Estimates were obtained using Stata statistical software.

**Figure 1:**
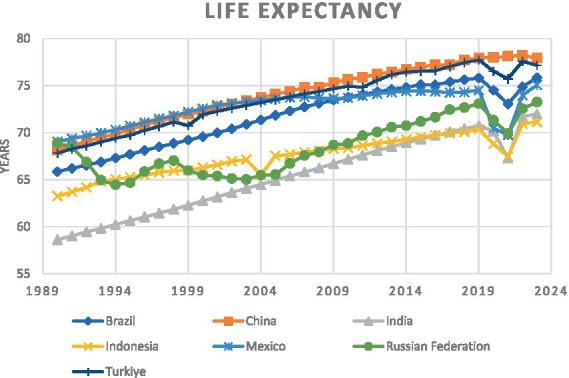
Annual trend of LE in E7 nations during the study span.

LE serves as the dependent variable, which has an average of 70.46 years, a median of 70.43 years, and a standard deviation of 4.28 years. In contrast with other variables, the small dispersion indicates that the gains in life expectancy have been steady for all countries in the E7 grouping. The range between the minimum and maximum of 58.62 and 78.20 years shows clear progress in improving population health during the entire analysis period. The negative skewness of −0.354 implies a slight clustering of data points at higher values of life expectancy. This is mainly due to the positive long-term trend in life expectancy seen for all economies. It can be easily visualized from Figure 1, showing how life expectancy is steadily increasing in all E7 countries through time in spite of temporary disruptions during the COVID-19 period. India has the most notable long-term improvement, going from less than 60 years in the early 1990s to more than 72 years by 2023, while China, Turkey, and Mexico have the highest life expectancy levels in recent years.

**Figure 2:**
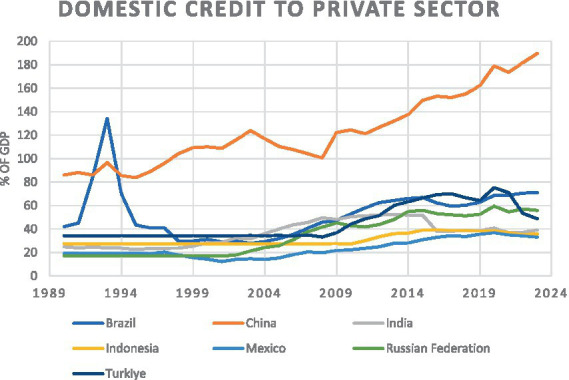
Annual trend of DCPS in E7 nations during the study span.

The level of DCPS, which can be used as a proxy for financial development, is highly variable, with an average level equal to 49.53% of GDP and an extremely high standard deviation of 35.67. The significant difference between the minimum and maximum values, being 12.24 and 189.61% respectively, indicates significant differences in financial sector development in the E7 countries. The positive skewness (1.843) and relatively high kurtosis (6.114) suggest that there were only a few cases when the observed value was very high for financial credit expansion. This statement is supported by Figure 2, which demonstrates a rather rapid growth in the level of domestic credit in China, especially in the early years of the 2000s, whereas other E7 countries had lower levels of financial deepening. The figure also reveals notable fluctuations in Brazil and Turkey, highlighting the uneven nature of financial sector development across emerging markets.

FDI has a mean value of 1.92% of GDP and a median of 1.82%, with a standard deviation of 1.36. These stats being so similar suggest that FDI was generally evenly spread in the sample. Still, the range from −2.76 to 6.16% highlights notable disparities among countries and over the years. As indicated by a skewness coefficient of 0.150 and kurtosis near the normal benchmark value of three, the distribution of FDI is fairly symmetrical compared to that of other variables in the sample. In summary, while FDI remained an important external financing source, there was still notable variation across locations and time periods.

INF shows the most variability of all the factors. Its mean is 61.34%, yet the median is just 6.80%, hinting at lots of inflation spikes. With a huge standard deviation of 284.98%, a max value of 2947.73%, skewness of 7.430, and kurtosis of 63.929, we see many outliers and volatile periods. This pattern happens due to hyperinflation in some E7 nations early on in their economic changes. Also, the big gap between mean and median suggests that inflation was typically mild, but it got hit by extreme economic shocks once in a while.

**Figure 3:**
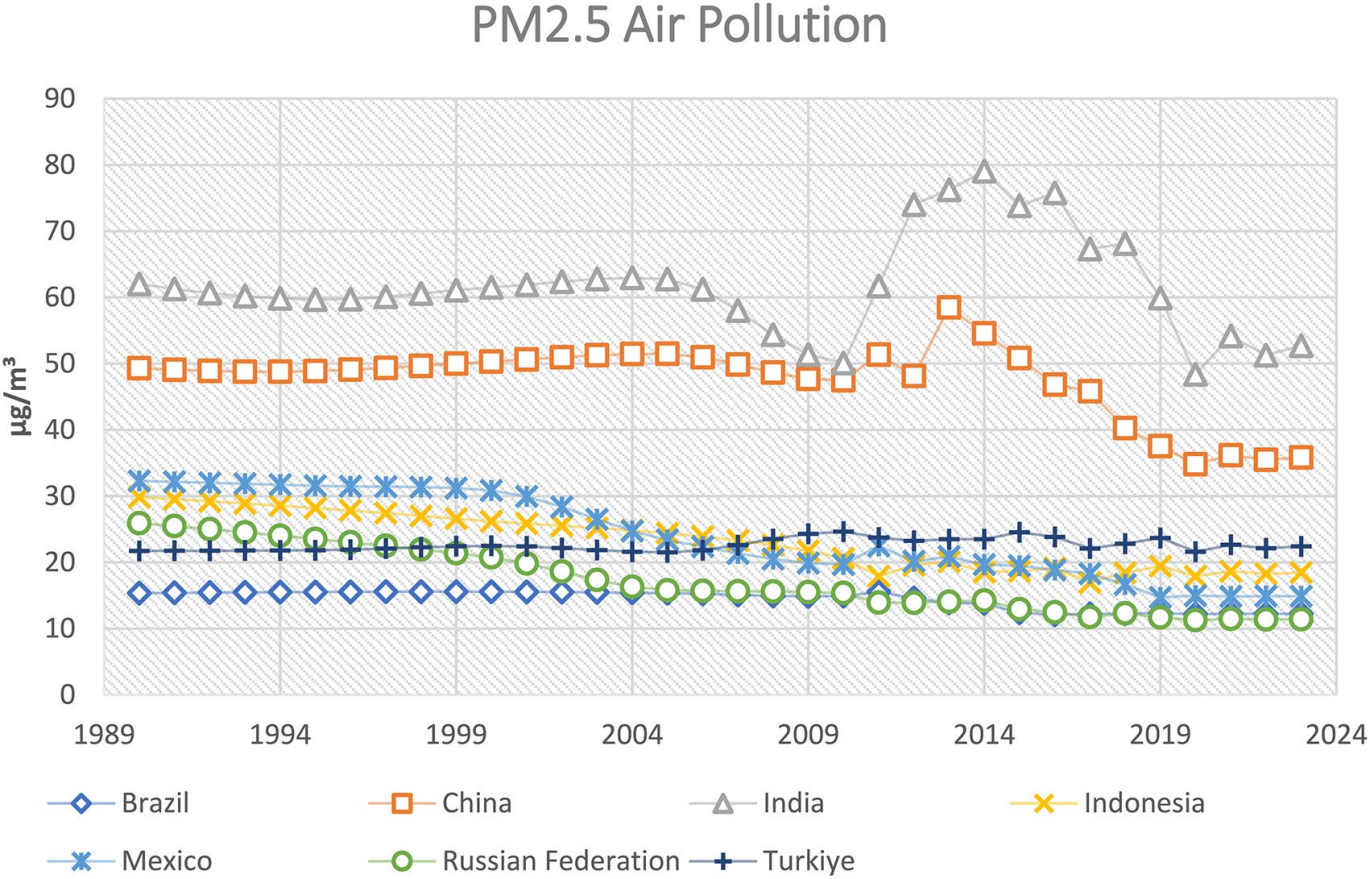
Annual trend of PM2.5 in E7 nations during the study span.

The average PM2.5 air pollution level is 30.11 μg/m^3^, ranging from 11.27 μg/m^3^ to 79.04 μg/m^3^. The PM2.5 data has a positive skewness of 1.006, meaning some areas have much higher pollution. Also, the standard deviation of 17.04 indicates big differences in environmental conditions across countries. Figure 3 really drives this home. It shows India with the highest PM2.5 for most of the observation period. China’s levels were also quite high early on but dipped later. Compared to that, Brazil and Russia had lower pollution. There’s a clear drop in China’s PM2.5 exposure after the mid-2010s. This suggests their efforts to manage the environment and clean up air quality are working. Overall, there are major differences in air quality issues among the E7 economies.

IMM has an average of 86.92 percent, with a median of 90 percent. The low standard deviation of 12.20 reinforces this consistency. With skewness at −1.078, most data points sit on the higher end, showing strong progress in immunization rates. Plus, the highest recorded figure is 99 percent, showing that some countries reached nearly universal vaccination at times during the study period. In all, these stats point to major advancements in preventive health services in the E7 economies over time.

**Figure 4:**
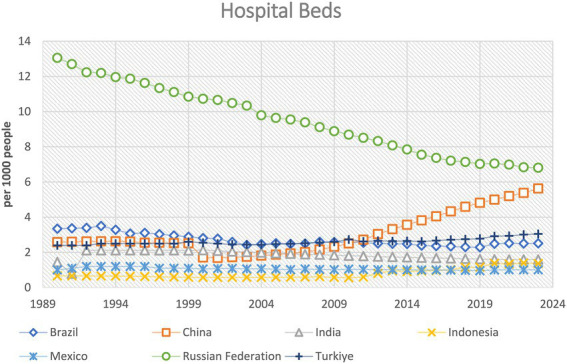
Annual trend of HBEDS in E7 nations during the study span.

HBEDS averages at 3.08 with a median of 2.40, showing considerable variation in health care setup between nations. Also, the big standard deviation with a value of 2.88, combined with the wide range from a minimum of 0.56 to a maximum of 13.06, highlights significant differences in health care infrastructure in E7 countries. Plus, with a high skewness of 1.902 and kurtosis of 5.717, it’s clear that only a few places have an unusually large amount of hospital bed infrastructure. This phenomenon can be noticed from Figure 4, where Russia continuously records the highest value for hospital beds, albeit with a declining trend. On the other hand, China experienced significant growth in its health care infrastructure after the year 2010. Most of the remaining E7 economies display relatively stable hospital bed capacity, although at substantially lower levels than Russia.

The results of the Jarque-Bera test reveal that the majority of the variables fail to follow the normal distribution. Specifically, DCPS, INF, PM2.5, IMM, and HBEDS significantly deviate from normality due to different levels of skewness and kurtosis. The life expectancy variable does not follow the normal distribution; at least, the distribution is much closer to normality than that of the other independent variables. On the other hand, FDI demonstrates distributional properties relatively similar to the normal distribution. Distributional properties of such nature are very common in both health and macroeconomic panel data sets and can be explained by different structures, the presence of outliers, and different development paths among the E7 countries. These features encourage the application of econometric methods intended for heterogeneous panel data environments.

The correlation coefficients between the variables used in the analysis can be seen in Table 3 and give an indication of the relationships between life expectancy and the factors that determine it within the E7 countries. Although correlations do not demonstrate causation, they help to determine the nature of the relationship between variables for the multivariate econometric analysis stage.

**Table 3:** Correlation matrix.

Variable	LE	DCPS	FDI	INF	PM2.5	IMM	HBEDS
LE	1.000						
DCPS	−0.528***	1.000					
FDI	0.376***	0.251***	1.000				
INF	−0.161**	0.030	−0.215***	1.000			
PM2.5	−0.268***	0.260***	−0.018	−0.131**	1.000		
IMM	0.691***	0.274***	0.364***	−0.075	−0.395***	1.000	
HBEDS	−0.110*	0.021	−0.118*	0.177***	−0.238***	0.311***	1.000

p*** < 1%, p* < 10%, and p** < 5%. Estimates were obtained using Stata statistical software.

There is a positive relationship between life expectancy (LE) and foreign direct investment (FDI) (*r* = 0.376, *p* < 0.01) as well as life expectancy (LE) and immunization coverage (IMM) (*r* = 0.691, *p* < 0.01). Considering the fact that life expectancy shows a relatively strong positive relationship with immunization coverage, it can be said that there is usually a high rate of life expectancy among countries where there are high rates of vaccination. In addition to this, the positive relationship between FDI and life expectancy suggests that more foreign investments usually accompany better population health outcomes.

Conversely, life expectancy is negatively related to DCPS (*r* = −0.528, *p* < 0.01), INF (*r* = −0.161, *p* < 0.05), PM2.5 exposure (*r* = −0.268, *p* < 0.01), and HBEDS (*r* = −0.110, *p* < 0.10). The negative relationship between life expectancy and PM2.5 exposure corresponds with the findings of the environmental-health literature, since air pollution is adversely associated with public health and contributes to premature deaths. Similarly, the negative relationship between life expectancy and inflation implies that inflation is associated with lower levels of life expectancy in nations facing higher inflation rates. On the contrary, it is rather counterintuitive that there is a negative relationship between life expectancy and DCPS since bivariate analysis does not take into consideration economic structure, institutional differences, levels of development, or any other confounders in these preliminary analyses. As a result, it is not appropriate to consider these initial correlations as evidence of causal linkages.

The correlation matrix also highlights some significant relationships between explanatory variables. Domestic credit to the private sector has a positive correlation with FDI (*r* = 0.251, *p* < 0.01), PM2.5 exposure (*r* = 0.260, *p* < 0.01), and immunization coverage (*r* = 0.274, *p* < 0.01). This indicates that the more advanced the financial development is, the more foreign investments, immunization rates, and air pollution there are in the region under consideration. Besides, FDI is positively correlated with immunization coverage (*r* = 0.364, *p* < 0.01); thus, countries that receive higher amounts of foreign investments also have higher rates of immunization.

Higher PM2.5 pollution levels are negatively correlated with immunization rates (*r* = −0.395, *p* < 0.01) and the number of hospital beds (*r* = −0.238, *p* < 0.01). In other words, it means that countries that have high pollution rates have low rates in terms of these two health indicators. In addition, there is a positive correlation between hospital beds and immunization coverage (*r* = 0.311, *p* < 0.01), indicating that these two indicators of healthcare-system capacity and preventive healthcare tend to move together across the E7 economies.

Generally, however, the correlation coefficients have moderate values and do not exceed levels where one would worry about extreme multicollinearity problems. This indicates that the variables measure different aspects of financial development, macroeconomic environment, environmental quality, health infrastructure, and people’s health. As such, the correlation test serves to provide evidence in favor of the empirical model, which requires further analysis using cointegration and long-run estimation methods.

According to Pesaran (49), the Cross-Sectional Dependency (CD) test results are shown in Table 4. They indicate significant reliance among E7 economies; the null hypothesis of independence was rejected for all variables at conventional significance levels.

**Table 4:** CD test.

Variable	Coeff.	*p*-value
LE	23.00***	0.000
DCPS	17.15***	0.000
FDI	5.48***	0.000
INF	9.59***	0.000
PM2.5	7.73***	0.000
IMM	11.13***	0.000
HBEDS	2.16**	0.025

p*** < 1% and p** < 5%. Estimates were obtained using Stata statistical software.

In particular‚ really high CD stats were seen in LE and DCPS‚ suggesting that financial development and health are affected by a lot of the same global factors․ Further analysis also reveals and tests for cross-sectional dependence for FDI‚ inflation‚ PM2․5 exposure‚ and IMM and HBEDS‚ highlighting the interconnectedness of these economic‚ environmental‚ and health variables across the countries under study․

The research shows that things like trade, money moves, tech spread, and global health issues can shock one E7 economy and then hit others too. So first-gen panel methods will not work here. This calls for using second-gen tests – panel unit root and cointegration checks, along with strong estimators such as CCEMG and Driscoll-Kraay errors. These tools handle how countries affect each other across the board, making them perfect for dealing with cross-sectional dependencies.

The Pesaran and Yamagata (50) slope homogeneity test results are shown in Table 5. The null hypothesis of slope homogeneity is rejected since both the Delta and adjusted Delta statistics are positive and statistically significant at the 1% level.

**Table 5:** Slope homogeneity test.

Stats	Coeff.	*p*-value
Delta	10.858***	0.000
Adj. delta	12.362***	0.000

p*** < 1%. Estimates were obtained using Stata statistical software.

These results show that life expectancy is not equally affected by financial development, foreign direct investment, inflation, environmental quality, and healthcare-related factors in each of the E7 economies. Given the significant variations in the sample countries’ healthcare systems, institutional quality, economic growth, environmental circumstances, and demographics, this outcome is to be expected.

Slope heterogeneity indicates that the underlying relationships may be oversimplified if a common coefficient is assumed for all countries. Therefore, estimators that can account for diverse country-specific effects, like the Common Correlated Effects Mean Group (CCEMG) estimator, are used as robustness tests to make sure the empirical results are reliable.

Stationarity features should be determined before analyzing the variables’ long-term association. As shown in Table 4, since the variables show cross-sectional dependence, the use of second-generation panel unit root tests is considered. The Cross-Sectionally Augmented IPS (CIPS) and Cross-Sectionally Augmented Dickey-Fuller (CADF) tests developed by Pesaran (51), which explicitly consider cross-sectional dependence, are used in this study due to their superiority over first-generation panel unit root tests.

The findings presented in Table 6 show mixed results on stationarity at the level. As per the CIPS tests, variables such as LE, DCPS, PM2.5, and HBEDS do not reject the null hypothesis of unit root, while FDI, INF, and IMM are found to be stationary at levels. In a similar vein, the CADF test shows that FDI, IMM, and HBEDS are stationary at levels, but LE, DCPS, INF, and PM2.5 are non-stationary at levels. Mixed results on the variables’ stationarity at levels are not uncommon and can be seen frequently in the case of macro-economic, environmental, and health variables. The mixed results at the levels show that the variables display different levels of persistence over time, which is typical of many macroeconomic, environmental, and health-related variables.

**Table 6:** Unit root test.

Variable	CIPS		CADF	
	Level	1st difference	Level	1st difference
LE	−2.732*	−5.519***	−2.541 (0.273)	−3.848*** (0.000)
DCPS	−1.877	−4.174***	−2.511 (0.303)	−3.412*** (0.001)
FDI	−3.691***	−5.722***	−3.535*** (0.000)	−4.773*** (0.000)
INF	−3.572***	−5.928***	−2.669 (0.161)	−4.685*** (0.000)
PM2.5	−1.907	−5.611***	−1.465 (0.996)	−4.376*** (0.000)
IMM	−4.132***	−5.828***	−2.911** (0.043)	−4.786*** (0.000)
HBEDS	−2.596	−5.826***	−3.235*** (0.004)	−4.399*** (0.000)

All specifications include a constant and linear trend. The maximum lag length was set to 1. For the CIPS test, the relevant critical values are −2.73 (10%), −2.84 (5%), and −3.06 (1%). p*** < 1%, p** < 5%, and *p < 10%. Estimates were obtained using Stata statistical software.

Following the first differencing procedure, the null hypothesis of a unit root is rejected for all variables using both the CIPS and CADF tests at the 1 percent level of significance. The above finding suggests that the non-stationary time series becomes stationary after applying the differencing procedure and thus meets the criteria necessary for testing the existence of cointegration in a panel data set. The results support the notion that macroeconomic, financial, environmental, and health variables frequently show long-term trends and gradually change over time.

In general, the unit root test outcomes demonstrate enough evidence that all variables have the same stochastic characteristics in order to conduct panel cointegration tests afterwards. It is important to identify the order of integration since any cointegration test must rely on variables having a stochastic characteristic of the same order. Therefore, the following stage is to find out whether life expectancy, along with related factors, shares a long-term equilibrium relationship among the E7 countries.

After ensuring the variables are stationary, we check if a long-run equilibrium relationship exists between life expectancy, financial development, foreign direct investment, inflation, PM2.5 exposure, immunization coverage, and healthcare infrastructure in E7 economies. We use the Pedroni and Kao panel cointegration tests for solid results, which are shown in Table 7.

**Table 7:** Panel cointegration test.

Test type	Statistic	*p*-value	Result
Pedroni test			
Modified Phillips–Perron *t*	1.9047**	0.028	Cointegration
Phillips–Perron *t*	−2.396***	0.008	Cointegration
Augmented Dickey–Fuller *t*	−3.006***	0.001	Cointegration
Kao test			
Modified Dickey–Fuller *t*	−6.305***	0.000	Cointegration
Dickey–Fuller *t*	−4.395***	0.000	Cointegration
Augmented Dickey–Fuller *t*	−3.732***	0.000	Cointegration
Unadjusted Modified Dickey–Fuller *t*	−7.500***	0.000	Cointegration
Unadjusted Dickey–Fuller *t*	−4.631***	0.000	Cointegration

p*** < 1% and p** < 5%. Estimates were obtained using Stata statistical software.

The Pedroni test results all suggest cointegration․ The Modified Phillips-Perron test is meaningful at the 5% level‚ while the Phillips-Perron and the Augmented Dickey-Fuller test statistics are meaningful at the 1% level. As the null hypothesis of no cointegration is rejected for all of the calculated statistics‚ the variables show a long-run relationship among them.

This is confirmed by the results of the Kao test: the Modified Dickey-Fuller‚ Dickey-Fuller, and Augmented Dickey-Fuller statistics and unadjusted *t* statistics are all negative and significant at the 1% level. The null hypothesis is always rejected‚ indicating that the variables are moving together over time and following the same long-run equilibrium path.

So, the Pedroni and Kao outcomes show that even though there are short-term wobbles, things like life expectancy, financial development, foreign investment, inflation, environmental quality, immunization coverage, and healthcare infrastructure stay pretty stable in the long run across the E7 economies. These findings justify the use of long-run estimators such as FMOLS and DOLS to quantify the magnitude and direction of these relationships.

Further evidence on the long-run equilibrium relationship will be obtained using the cointegration test of Westerlund (53). In contrast to residual-based cointegration tests, the cointegration test of Westerlund assesses the presence of an error correction model. It is worth noting that Westerlund’s test works well in panel datasets with cross-section dependence and heterogeneity. The results are shown in Table 8.

**Table 8:** Westerlund cointegration test.

Statistic	Value	*Z*-value	*p*-value
Gt	−7.295***	−12.275	0.000
Ga	−32.319***	−4.537	0.000
Pt	−19.589***	−11.864	0.000
Pa	−30.611***	−5.082	0.000

p*** < 1%. Reported *p*-values are asymptotic *p*-values based on the standard normal approximation and are not bootstrap-based. Estimates were obtained using Stata statistical software.

All four Westerlund cointegration test statistics, Gt, Ga, Pt, and Pa, are significant at the 1% significance level. As such, the null hypothesis of no cointegration is rejected regardless of whether it is the group mean test statistic or the panel test statistic. The significance of both group mean test statistics and panel test statistics signifies the existence of a long-run equilibrium in the variables of interest, both at the individual-country level and for the panel as a whole.

It is important to note that the *p*-values calculated above are the asymptotic *p*-values, using the standard normal distribution, not bootstrap inference. However, it is worthwhile to point out that the results from the Westerlund approach are completely in line with those obtained by conducting cointegration tests using both the Pedroni and Kao approaches, thus lending further credence to the existence of a long-term relationship between life expectancy and its underlying factors in the E7 countries.

Generally, the findings from the Pedroni, Kao, and Westerlund tests enhance the likelihood of panel cointegration, making it feasible to estimate long-run coefficients using the FMOLS and DOLS techniques. Consequently, the application of long-run techniques such as FMOLS and DOLS is justified when investigating the relationships among financial development, FDI, inflation, PM2.5 pollution, healthcare variables, and life expectancy.

Long-run coefficient values using the FMOLS and DOLS estimation methods are shown in Table 9. Consistency in results from both estimation methods increases the reliability of the results because the FMOLS and DOLS methods use different methodologies to deal with endogeneity and serial correlation issues associated with cointegration in panels. Generally, the results show that there is stability in long-run relationships among financial development, foreign investment, macroeconomic variables, environmental quality, health-related variables, and life expectancy in E7 countries. In most instances, the estimated coefficients have a consistent sign and significance level across the two methods.

**Table 9:** Panel FMOLS and DOLS estimates.

Variable	FMOLS		DOLS	
	Coefficient	*t*-Statistic	Coefficient	*t*-Statistic
DCPS	0.12***	14.71	0.05***	12.56
FDI	0.05***	4.37	0.08***	6.89
INF	−2.94***	−19.12	−3.39***	−11.85
PM2.5	−0.03***	−13.12	−0.90***	−14.78
IMM	0.21***	43.92	0.05	1.55
HBEDS	−1.77***	−10.03	−7.15***	−14.19
Countries	7	-	7	-
Observations	238	-	238	-
Period	1990–2023	-	1990–2023	-
Cointegration	Confirmed	-	Confirmed	-
Country effects	Yes	-	Yes	-
Time effects	No	-	No	-
Trend	No	-	No	-
Lead–Lag structure	-	-	(1,1)	-

Dependent variable: LE. ***p < 1%. All variables (including the dependent variable) were transformed to z-scores before estimation. Estimates were acquired using Stata statistical software.

Domestic credit to the private sector is positively associated with life expectancy and statistically significant under FMOLS and DOLS methodologies. In the former case, the coefficient is 0.12 (*t* = 14.71, *p* < 0.01); in the latter, the coefficient is 0.05 (*t* = 12.56, *p* < 0.01). As the data have been standardized before estimation, it could be concluded that there exists a positive long-term relationship between financial development and life expectancy. It corresponds to the expectations stated in Hypothesis 1 and aligns with Grossman’s theory of health capital, which postulates the possibility for financial resources to make people engage in health-increasing activities. Such a conclusion is supported by the results of numerous empirical studies, such as those of Mbodj and Laye (16), Miar (17), and Jalili et al. (18), which report positive associations between financial development and population health outcomes. In the context of the E7 economies, the findings suggest that periods characterized by greater financial deepening tend to coincide with higher levels of life expectancy.

Foreign direct investment (FDI) is similarly positively correlated and significantly associated with life expectancy. The respective FMOLS and DOLS estimates are 0.05 (*t* = 4.37, *p* < 0.01) and 0.08 (*t* = 6.89, *p* < 0.01). These findings are aligned with Hypothesis 2 as well as with the general predictions of Modernization Theory about foreign investments leading to the diffusion of technology and economic growth. Although it should be stated that the current analysis cannot make a claim about causality, the positive correlation coefficients reveal that increased foreign investments are usually correlated with increased life expectancy over the long run. This conclusion supports the findings of several recent studies conducted by Zeeshan and Singh (21), Nguyen et al. (22), and Lara-Llanderal et al. (23).

INF shows an inverse and significant relationship with life expectancy in both models. In the case of FMOLS, the coefficient of inflation (−2.94) is statistically significant at *t* = −19.12, *p* < 0.01, whereas for DOLS, it is significant at −3.39, *t* = −11.85, *p* < 0.01. These results are in agreement with Hypothesis 3 as well as the theoretical literature that highlights the significance of macroeconomic stability as a critical requirement for societal well-being and health. Inverse signs of the coefficients suggest a negative relationship between inflation and life expectancy in the E7 nations. The findings are in accordance with past empirical studies carried out by Pappas and Boukas (28), Manamperi et al. (29), and Islam et al. (30), who document adverse associations between inflation and various indicators of well-being.

Exposure to PM2.5 pollutants is negatively and significantly associated with life expectancy in both estimators. For the FMOLS estimator, the coefficient is −0.03 (*t* = −13.12, *p* < 0.01); for the DOLS estimator, the coefficient is −0.90 (*t* = −14.78, *p* < 0.01). The empirical findings lend support to Hypothesis 4 and fit well within the Environmental Health Risk Framework that underscores the impact of environmental factors on health conditions. The negative coefficients imply that an increase in PM2.5 exposure is likely to be accompanied by decreases in life expectancy in the long term. The empirical results are in line with previous findings, such as those reported by Henning (35), Weber et al. (36), and Zhang et al. (37), concerning the adverse health effects of air pollution. From the environmental-health perspective, the empirical findings illustrate the importance of air quality as one of the factors that influence population health in the E7 economies.

The results regarding immunization coverage (IMM) are slightly conflicting. According to the results of the FMOLS regression analysis, IMM is positively correlated with life expectancy, and the correlation is statistically significant (coefficient = 0.21; *t* = 43.92; *p* < 0.01). In contrast, the coefficient obtained in the DOLS model is also positive (coefficient = 0.05), but it is statistically insignificant (*t* = 1.55). Consequently, Hypothesis 5 gets some support from this research. Generally speaking, the positive signs of the coefficients obtained are in line with the Health Production Theory. They are also in agreement with other studies, which point out that effective immunization campaigns are crucial for good population health outcomes (41, 44). However, the variation in statistical significance across estimators suggests that the strength of this long-run association may be sensitive to model specification.

The regression results for hospital beds per 1,000 people (HBEDS) do not confirm the theoretical predictions suggested in Hypothesis 6. The coefficients have negative signs and are statistically significant for both FMOLS (−1.77; *t* = −10.03; *p* < 0.01) and DOLS (−7.15; *t* = −14.19; *p* < 0.01). Hence, Hypothesis 6 is rejected in light of the empirical evidence provided in this study. Despite being contradictory to most of the available studies, the negative coefficient cannot be viewed as a confirmation of the assumption that the availability of healthcare resources negatively affects life expectancy. In fact, the negative sign might be related to the fact that hospital bed numbers serve as an indicator of healthcare demand, disease prevalence rates, demography, and other characteristics that are embedded in healthcare infrastructure. However, since the number of hospital beds is but one component of healthcare infrastructure and cannot be used as a proxy for measuring healthcare quality, efficiency, accessibility, and prevention efforts, the observed relationship should be interpreted as a long-run statistical association rather than a causal effect.

Generally, the findings indicate that there is confirmation of H1, H2, H3, and H4 hypotheses, partial confirmation of H5, and refutation of H6. The consistency in the estimated values by FMOLS and DOLS increases the probability of obtaining stable long-run relationships. Therefore, the results imply that life expectancy within the E7 countries is correlated not just with factors relating to the healthcare sector but also with the financial, macroeconomic, and environmental determinants. Specifically, a more developed financial sector and foreign investment are positively linked with life expectancy, while higher inflation rates and increased pollution in terms of PM2.5 are negatively correlated with life expectancy. These findings underscore the importance of considering economic, environmental, and healthcare-related factors jointly when examining long-term population health outcomes.

Diagnostic tests were carried out to evaluate the suitability of the panel specification and the features of the error structure before doing the robustness analysis. Table 10 presents the findings.

**Table 10:** Diagnostic tests.

Test	Statistics	Prob. value
White’s test	129.84***	0.000
Hausman test	94.48***	0. 000

p*** < 1%. All variables were transformed to *z*-scores before estimation. Estimates were obtained using Stata statistical software.

Heteroskedasticity in the model is indicated by the highly significant White’s test statistic (129.84, *p* < 0.01). This result implies that the variance of the error terms is not constant across observations, which is a common characteristic of macro-panel data, including nations with varying levels of development, institutional frameworks, and economic sizes. In order to achieve trustworthy statistical inference, robust estimating procedures are justified when heteroskedasticity is present.

The null hypothesis that the random-effects estimator is consistent is rejected as a result of the Hausman test’s extremely significant result (94.48, *p* < 0.01). The fixed-effects assumption is more suitable for the analysis because this result shows that country-specific effects are associated with the explanatory factors. As a result, the fixed-effects model is used for the ensuing robustness evaluation, and Driscoll–Kraay standard errors are used to take cross-sectional dependence, serial correlation, and heteroskedasticity into consideration.

In order to evaluate the robustness of the initial results of the FMOLS and DOLS models, further estimations will be undertaken using the fixed effects regression model with the Driscoll-Kraay standard errors (FE-DKSE) and the Common Correlated Effects Mean Group estimator (CCEMG). Their results are provided in Table 11. This is justified by the findings of the presence of cross-sectional dependency and slope heterogeneity from the diagnostic tests. The FE-DKSE estimator yields standard errors that are robust to heteroskedasticity, serial correlation, and cross-sectional dependency, while the CCEMG estimator further considers the existence of unobserved common factors and slope heterogeneity coefficients across countries.

**Table 11:** Robustness check.

Variable	FE-DKSE		CCEMG	
	Coefficient	DK std. error	Coefficient	Std. error
DCPS	0.697***	0.106	0.009	0.335
FDI	0.083	0.061	−0.015	0.013
INF	−0.122**	0.051	−0.088	0.455
PM2.5	−0.062	0.094	−0.118	0.073
IMM	0.409***	0.046	0.001	0.024
HBEDS	−0.140	0.210	−0.031	0.142
Constant	−2.03E-09	0.051	0.473***	0.075
Number of obs.	238	-	238	-
Number of groups	7	-	7	-
F-stat./Wald chi^2^	189.70	-	14.08**	-
R-sq.	0.683	-	-	-
Max. lags	3	-	-	-
Root mean squared error	-	-	0.074	-

Dependent variable: LE. ***, *, * indicate significance at 1, 5, and 10% levels, respectively. All variables (including the dependent variable) were transformed to z-scores before estimation. Estimates were obtained using Stata statistical software.

The findings obtained using the FE-DKSE are consistent with several patterns identified in the baseline analysis. First, domestic credit to the private sector (DCPS) is found to have a statistically significant positive relationship with life expectancy, with a coefficient of 0.697 (*p* < 0.01). In addition, immunization coverage (IMM) is still found to have a positive statistically significant coefficient with life expectancy, with a coefficient value of 0.409 (*p* < 0.01). Further, the variable inflation (INF) is seen to have a negative and statistically significant coefficient at the 5% level (*β* = −0.122, *p* < 0.05), implying that life expectancy is negatively related to inflation. Conversely, the variables FDI, PM2.5, and hospital beds (HBEDS) are shown to retain similar coefficient signs as those obtained from the baseline models; however, the coefficients are not statistically significant in the FE-DKSE model. The model explains about 68.3% of within-country variation in life expectancy (R^2^ = 0.683), indicating a relatively strong overall fit.

Further analysis is gained through the CCEMG estimation by letting the effects of the explanatory variables differ among countries while holding constant the unobservable common factors. Generally speaking, the coefficient signs derived from the CCEMG estimation are maintained compared to the signs in the benchmark FMOLS and DOLS estimations, such that DCPS and IMM remain positive toward the life expectancy variable, while inflation, PM2.5 levels, and HBEDS maintain their negative coefficient signs. Nevertheless, the majority of the coefficients do not turn out to be significant in terms of their statistical relationship in the CCEMG model, suggesting that the relationship between the variables changes among different nations.

One exception to this is the foreign direct investment (FDI), whose coefficient becomes negative in the CCEMG estimation while it was positive in the FMOLS, DOLS, and FE-DKSE estimators. Although the magnitude of this coefficient is relatively low, this change in sign could possibly signify that the effect of foreign investment on life expectancy is not always the same among the E7 countries. This implies that the relationship between FDI and health outcomes can be contingent upon a country’s unique institutions, economic, and social circumstances, all of which have been appropriately considered in the CCEMG approach.

Generally, the robustness test confirms some parts of the findings from the base model. The positive relationship between financial development, immunization, and life expectancy on one side and the negative relationship between inflation and life expectancy on the other side remains evident in the robustness estimations. In relation to PM2.5 concentration and number of hospital beds, there appears to be some consistency in coefficient signs in all estimations, even though in some cases the findings are statistically insignificant in the robustness estimations. In general, however, the findings demonstrate that the key long-run relationships discovered in the base model estimates are generally robust, but they show the importance of controlling for cross-sectional dependence and common factor, and heterogeneous country responses when interpreting the magnitude and significance of the estimated relationships.

## 5 Conclusion and policy recommendations

### 5.1 Conclusion

The current research investigates the relationships between life expectancy and several financial, economic, environmental, and health variables in the E7 countries for the period from 1990 to 2023. These variables include domestic credit to the private sector, foreign direct investment, inflation rate, PM2.5 exposure, immunization rate, and number of hospital beds per 1,000 population. To conduct a rigorous empirical investigation, a robust methodology was utilized that consisted of cross-sectional dependence and slope heterogeneity tests, panel unit root tests (second generation, such as CIPS and CADF tests), panel cointegration tests (such as Pedroni, Kao, and Westerlund tests), and methods of estimating the long-run relationship between the dependent and independent variables, like FMOLS and DOLS. Additional robustness checks were conducted using fixed effects with Driscoll-Kraay standard errors (FE-DKSE) and the Common Correlated Effects Mean Group (CCEMG) estimator.

The results of the empirical analysis show the existence of a long-run relationship between life expectancy and its determinants, which include factors related to finance, economy, environment, and healthcare. Both FMOLS and DOLS estimations reveal that the private sector’s domestic credit and foreign direct investment have a positive relationship with life expectancy. However, at the same time, both inflation and PM2.5 exposure have negative relationships with life expectancy. This proves the necessity to consider financial and macroeconomic stability and the environment as factors determining the health of the populations. Vaccination has a positive relationship with life expectancy, despite the differences between the results depending on the estimations. On the other hand, the number of hospital beds shows a negative relationship with life expectancy, which means that this variable can determine broader healthcare-system characteristics, healthcare demand, or underlying disease burdens rather than healthcare capacity alone.

In terms of the linkage between environmental factors and public health issues, the results highlight the relevance of PM2.5 emissions as one of the critical determinants of longevity for populations of emerging economies. In this regard, the negative association between air pollution and life expectancy is aligned with the current trend in scientific literature concerning the negative association of air pollution with population health status and early mortality rates. Given the high speed of the development process in the E7 countries, environmental aspects still matter for long-term public-health and sustainable-development strategies.

Robustness checks confirm most of the key results from the baseline estimations. For the FE-DKSE model, domestic credit and immunization coverage remain positively and significantly associated with life expectancy, whereas inflation still shows a significant negative association. While PM2.5 concentration and hospital beds still have a negative sign on their coefficients, their significance is not preserved in all the robustness estimations. The CCEMG estimation shows a similar direction of effects on the associations, although the magnitudes and significances can change among countries by considering an unobserved common factor and heterogeneous responses. Among other things, the association of foreign direct investments with life expectancy becomes non-uniform in the E7 nations when using the CCEMG method.

Generally, it can be concluded that life expectancy in E7 countries is influenced by not only healthcare-related variables but also economic development, environment, and financial issues. The fact that all three types of long-term relationships have been observed via different estimation methods strengthens the results of the research conducted; however, at the same time, it underlines the importance of joint consideration of economic development, environment, and healthcare in assessing population health in E7 countries.

### 5.2 Policy recommendations

The results of this study indicate that life expectancy within the E7 countries can be linked to a number of economic, macroeconomic, environmental, and healthcare variables. Considering the presence of robust long-run relationships identified via the empirical analysis, policy considerations should be based on the economic variables that display a stable relationship with population health.

Financial development, first of all, is positively correlated with life expectancy according to the positive coefficients for domestic credit to the private sector in both the baseline regressions and the robustness tests. This result implies that there may be some policy measures that enhance financial inclusiveness, provide easier access to financial markets, and improve financial intermediation in general to contribute to a socioeconomic environment conducive to better health. Access to financial sources could help households allocate more funds for their healthcare needs and also spend on education and nutrition. Considering different levels of financial system development among E7 countries, individual approaches to financial system reform would be reasonable.

Secondly, the negative relationship between inflation and life expectancy clearly indicates the significance of macroeconomic stability. An increase in inflation implies a decrease in life expectancy in all baseline estimations, and even in the robustness estimation, there exists a negative relationship between the two. The results indicate that measures aimed at price stability can ensure that households do not lose their buying power and hence can afford better access to health care facilities, medicines, and nutritional foods. Furthermore, macroeconomic stability can aid the government in making sustainable investments in public-health programs and healthcare infrastructure over time.

Thirdly, the findings highlight the significance of environmental quality in the determination of the health status of populations in the long run. There is a significant negative correlation between exposure to PM2.5 and life expectancy based on the baseline estimates provided above. Indeed, air pollution has been recognized as a significant health threat in the environmental-health discourse. As such, measures aimed at the minimization of pollution by particulate matter, including emissions reduction, increased use of green energy, improved environmental monitoring, and sustainable development of industry and transport, may contribute to healthier living environments. In rapidly industrializing and urbanizing economies, environmental protection and public-health objectives should be viewed as closely interconnected policy priorities.

Fourthly, immunization coverage has a positive correlation with life expectancy; however, the degree of significance differs across different estimators. Still, the results imply that preventive healthcare is still an essential part of long-term population health policies. As such, decision-makers should consider having high immunization coverage rates and enhancing public health strategies geared toward disease prevention, particularly among vulnerable and underserved populations. Further investment in preventive healthcare will aid in achieving improvements in overall population health through the reduction of preventable diseases.

Findings concerning foreign direct investment call for a more in-depth interpretation. Although the positive connection between foreign direct investment and life expectancy is observed from the FMOLS, DOLS, and FE-DKSE estimates, from the CCEMG model, there seems to be evidence that this connection differs from one country to another. The heterogeneity of results implies that the link between foreign investment and the health of the populace is determined by country-specific institutional, economic, and social conditions. Thus, policies designed to encourage foreign investment will prove more successful if they are implemented together with policies that enhance governance, institutional quality, and the ability to direct investments into activities that improve social welfare and public health.

Last but not least, the negative correlation between hospital beds and life expectancy indicates that the healthcare system infrastructure must not be assessed merely in terms of physical capacity. Because hospital beds may indicate the need for healthcare services, demographic considerations, or the presence of illnesses, among others, the quality, effectiveness, efficiency, and provision of preventive care in the healthcare systems must be considered as well. The provision of more primary care services, an improved healthcare service delivery system, and better-quality medical care services must be considered as significant as healthcare infrastructures themselves. This consideration is particularly relevant for the E7 economies, where healthcare systems differ substantially in structure and stage of development.

In summary, the analysis indicates that better population health is bound to be linked with a coherent policy strategy that ensures development in the finance sector, economic stability, a healthy environment, preventive health measures, and an efficient healthcare system. As a whole, the results indicate that there is a need for recognizing economic, environmental, and health policies as complementary elements of long-term strategies aimed at supporting population well-being and longevity.

### 5.3 Limitations and future research

This study has several limitations that should be acknowledged. First, the analysis focuses on a selected set of financial, macroeconomic, environmental, and health-related variables, which, although relevant to the objective of the study, do not fully capture the broader institutional, environmental, and social determinants of health outcomes in emerging economies. Important factors such as public health expenditure, governance quality, education, economic growth, technological change, and income inequality were not incorporated and may also shape life expectancy across the E7. Second, the use of country-level panel data may conceal substantial within-country disparities, particularly in large and heterogeneous economies such as China, India, Brazil, and Indonesia, where regional inequalities in healthcare access and socio-economic conditions can be considerable. Third, although the study covers a long time span from 1990 to 2023, the aggregate panel framework is limited in its ability to fully isolate the effects of major structural shocks such as the COVID-19 pandemic, global financial crises, or recent inflationary surges. Fourth, while FMOLS and DOLS are appropriate for examining long-run relationships in cointegrated panels, they do not fully address all possible forms of cross-country heterogeneity and dynamic complexity. Future research may therefore extend this work by incorporating broader sets of institutional and environmental variables, using disaggregated or subnational data, and applying alternative panel estimators that better capture heterogeneity, endogeneity, and cross-sectional dependence. Comparative studies across other country blocs such as BRICS, G7, or N-11, as well as research focusing on post-pandemic health resilience and the role of digital health systems, would also provide useful directions for deepening understanding of the finance-health nexus in emerging economies.

## Data Availability

The original contributions presented in the study are included in the article/supplementary material, further inquiries can be directed to the corresponding author.

## Appendix A

**Table A1:** Summary of interpolated observations.

Variable	Country	Missing year(s)	No. of interpolated obs.
DCPS	Indonesia	2008	1
DCPS	Russian Federation	1990, 1991, 1992, 2000, 2023	5
FDI	Russian Federation	1990, 1991	2
IMM	Russian Federation	1990, 1991	2
INF	Russian Federation	1990, 1991, 1992	3
HBEDS	Brazil	1998, 2022	2
HBEDS	India	2023	1
HBEDS	Indonesia	1996	1
HBEDS	Mexico	2023	1
PM2.5	Brazil	2023	1
PM2.5	China	2023	1
PM2.5	India	2023	1
PM2.5	Indonesia	2023	1
PM2.5	Mexico	2023	1
PM2.5	Russian Federation	2023	1
PM2.5	Türkiye	2023	1
Total			25

The missing values were imputed by linear interpolation to maintain the balance of the panel data. A total of 25 values were imputed out of a total of 1,666 values (238 country-year observations × 7 variables). This amounts to about 1.5% of the total observations. The interpolated observations were concentrated in a small number of countries, years, and variables and, therefore, are not expected to materially affect the estimated long-run relationships.
